# Secondary Cicatricial Scalp Alopecia Caused by Brunsting-Perry Pemphigoid: A Case Report

**DOI:** 10.7759/cureus.83828

**Published:** 2025-05-10

**Authors:** Ihor Kohut, Antonina Kalmykova, Halyna Bezkorovayna

**Affiliations:** 1 Dermatology, Skin Health Center, Ternopil, UKR; 2 Dermatopathology, Pathology Laboratory (Experimental Pathology Laboratories), Kyiv, UKR

**Keywords:** brunsting-perry pemphigoid, bullous disease, cicatricial alopecia, dermoscopy, pemphigoid

## Abstract

Brunsting-Perry pemphigoid is a rare, chronically persistent bullous dermatosis, localized to the head, scalp, and neck, causing residual scars and cicatricial alopecia. Herein, we present a case of Brunsting-Perry pemphigoid in a 68-year-old Caucasian woman presenting with over 10 years of history of slowly progressing patchy cicatricial alopecia. Dermoscopy showed scarring, a milky red background, and a typical picture of a “fried-egg sign” representing specific follicular damage. A skin biopsy revealed a subepidermal blister with dermal inflammation. Immunopathology shows strong linear continuous deposits of C3c and IgM along the basement membrane, and moderate to weak linear reactions to IgG and IgA. Intralesional betamethasone was successful in the treatment, and topical mometasone furoate lotion was used to maintain the result. Our case suggests that Brunsting-Perry pemphigoid may be underdiagnosed as the reason for scarring alopecia, considering the scarce information about the disease in the literature.

## Introduction

Brunsting-Perry pemphigoid (BPP) is a rare, chronically persistent blistering localized to the head, scalp, and neck, causing residual scars and cicatricial alopecia [1,2]. BPP is primarily a disease of elderly and Caucasian individuals. Approximately 63 cases of BPP were reported in English-language literature from 1950 to July 2021 [3].

BPP is classified within the mucous membrane pemphigoid (MMP) spectrum. However, mucosal manifestation is scarce or mild, and skin atrophy and scar formation are the leading signs in the clinical picture. Histopathology presents a subepidermal blister with a dermal inflammatory infiltrate, often eosinophilic. Immunopathologic examination shows IgG and often C3 deposits linearly distributed along the basement membrane zone [3]. This case was previously presented as an abstract at the Innovations in Dermatology: Spring Conference 2021.

## Case presentation

**Figure 1:**
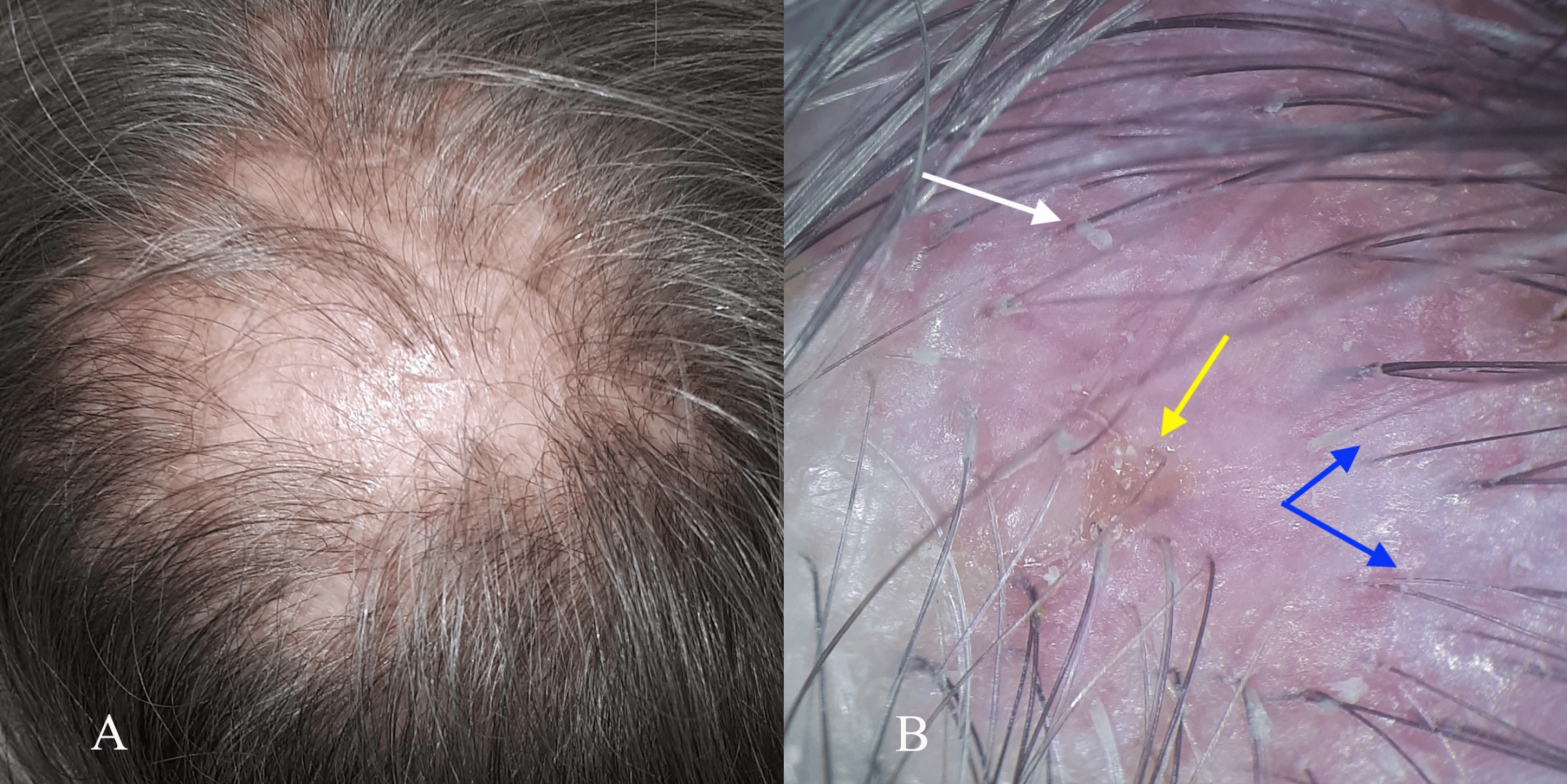
Clinical presentation of cicatricial alopecia caused by Brunsting-Perry pemphigoid A) Multifocal round/oval 10-25 mm moderately atrophic scar-like areas of hair loss without follicles in the temporal and parietal areas. B) Dermoscopy shows pink plaques with white scales (white arrow) and small yellow crusts (yellow arrow). Hairs of the inflamed areas are thin and single and may be easily pulled out (blue arrows).

A 68-year-old Caucasian woman presented with over 10 years of history of slowly progressing patchy scalp hair loss. Physical examination of temporal and parietal areas reveals multifocal moderately atrophic scar-like areas of hair loss, merged on the vertex (Figure 1A), pink plaques with white scales, yellow crusts over follicular openings, and thin and single hair (Figure 1B).

**Figure 2:**
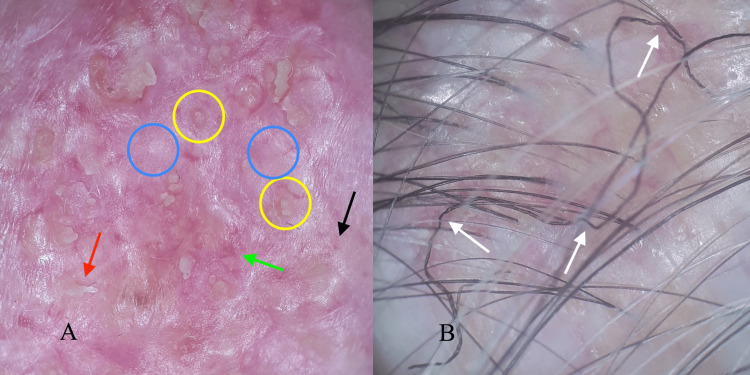
Dermoscopy presentation of cicatricial alopecia caused by Brunsting-Perry pemphigoid A) White scarring interfollicular confluent areas on a milky red background with absent follicular openings, presenting fibrosis (blue circles). Yellow dots with a whitish halo (“fried-egg sign”) correspond to follicular openings covered by residues of detached epidermis (yellow circles). White thick polygonal scales with protruding edges represent epidermolysis of the interfollicular area (red arrow). Elongated serpentine (green arrow) and dotted vessels (black arrow) display an inflammation; B) Few hairs emerge in the cicatricial area, some shafts are irregularly angulated (white arrows).

Dermoscopy shows white scarring areas on a milky red background, absent follicular openings, white scales, serpentine and dotted vessels, yellow dots with a whitish halo (“fried-egg sign”) around follicular openings (Figure 2A), few thin, irregularly angulated hairs (Figure 2B).

Therefore, it has been suggested that white scarring represents fibrosis, white lamellar scales appear for interfollicular epidermolysis, yellow dots illustrate detached epidermis of follicular openings, and thin and angulated hairs mark dystrophy in cicatricial areas. Unopened blisters are rare to be captured by dermoscopy.

**Figure 3:**
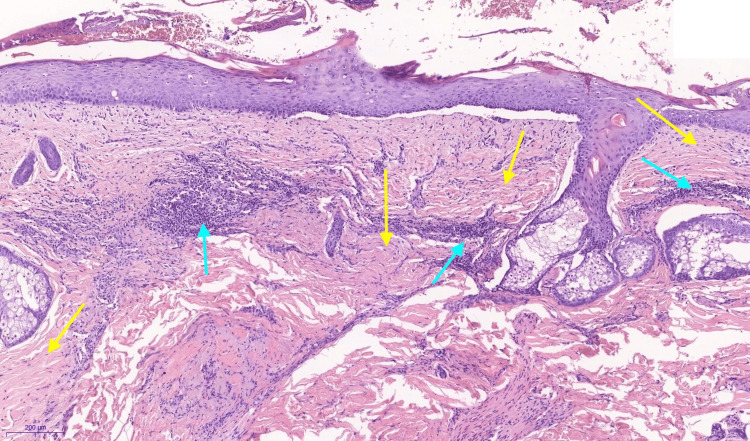
Histopathology presentation of cicatricial alopecia caused by Brunsting-Perry pemphigoid Marked cicatricial (yellow arrows)changes with moderate diffuse and perivascular lymphohistiocytic infiltrate (blue arrows) are seen in the dermis. Some cleft formations along the basement membrane can be noted.

Histopathology showed hyperorthokeratosis, slight acanthosis, tendency for subepidermal cleft formation, cicatricial changes in the dermis and in the places of pre-existing follicles, dense perivascular and moderate diffuse lymphohistiocytic infiltrate, admixture of plasma cells; also, foci of solar elastosis were noted under the scars. These histopathological findings are not highly specific separately, but along with the clinical manifestation, they can be suggestive of a diagnosis of BPP (Figure 3).

**Figure 4:**
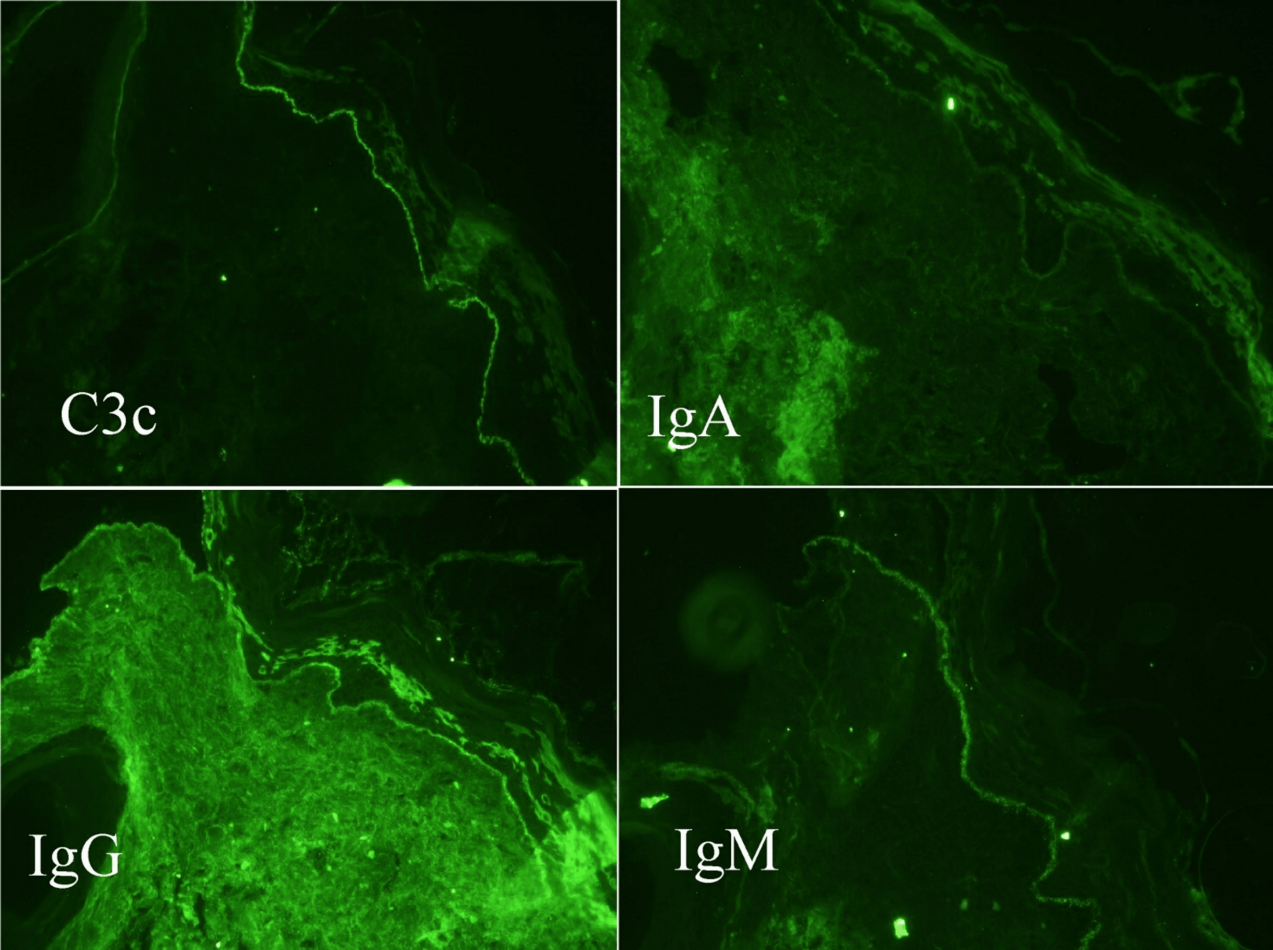
Direct immunofluorescence microscopy findings of cicatricial alopecia caused by Brunsting-Perry pemphigoid Linear deposits of C3c and IgM along the epidermal basement membrane can be appreciated. IgA and IgG deposits were barely seen and seem to represent non-specific reactions.

Direct immunofluorescence microscopy revealed strong linear continuous deposits of C3c along the basement membrane, linear and granular deposits of IgM, and moderate to weak linear reactions to IgG and IgA. These findings support the diagnosis of BPP in the appropriate clinical context, along with the histopathological findings (Figure 4).

Intralesional betamethasone suspension injections (betamethasone dipropionate equivalent to 5 mg/ml and betamethasone sodium phosphate equivalent to 2 mg/ml) were used every three weeks, four consecutive times. The dose used for intralesional injections did not exceed 7 mg of betamethasone for a one visit. After the course of treatment, erosions were absent, yellow dots and white scales became rare, and no new scarred areas were detected. Topical mometasone furoate lotion 2-3 times a week was effective in preventing a relapse of the disease during a year of supervision.

## Discussion

Differential diagnosis of the BPP can be challenging and includes bullous pemphigoid (BP), lichen planopilaris, pseudopelade of Brocq, erosive pustular dermatosis of the scalp, giant cell arteritis, epidermolysis bullosa acquisita (EBA), dermatitis artefacta, dermatitis herpetiformis, linear IgA bullous dermatosis, bullous systemic lupus erythematosus, and bullous impetigo [4-6].

Originally, BPP was reported by the authors as a variant of “benign pemphigoid.” Based on immunofluorescent findings, BPP represented a blistering disorder distinct from bullous pemphigoid and was reclassified as the cutaneous variant of MMP. Additionally, scarring is considered a characteristic sign for a BPP, MMP, and EBA but not for a BP [3].

Direct immunofluorescence of BPP exhibits a deposition of IgG and IgA linearly throughout the length of the basement membrane, as well as IgM, C3c, and C3d [7]. Recently, immunohistochemical examinations showed strong linear C4d deposits along the basement membrane zone. [8]. In salt-split BPP skin specimens, IgG and C3 are localized on the epidermal side only [9] or may be present along both the dermal and the epidermal surfaces [10]. The extrafollicular and follicular basement membrane may linearly deposit IgG4 [11].

According to immunohistology findings, BPP is considered to be presented as a variant of pemphigoid, EBA, or intermediate. In the Brunsting-Perry variant of bullous pemphigoid, the immune deposits of BP180 and epidermolysis are observed in the lamina lucida, particularly underneath the hemidesmosomes. A Brunsting-Perry variant of EBA is suggested when blister cleavage and immune deposition are both located at the sub-lamina densa level. Intermediate form between BP and EBA is distinguished when blister splitting is located intra a lamina lucida but immunoglobulin G deposition is along the lamina densa [12]. If collagen VII autoantibodies are detected, a diagnosis of the Brunsting-Perry variant of EBA can be made [4,8].

BPP has distinctly different clinical manifestations from MMP. However, BPP is also associated with the autoantibodies to antigens of BP and MMP, such as BP180 (NC16A and C-terminal domains), BP230, LAD-1, and laminin-332 [2,4,7]. In the case of BPP in a middle-aged male, IgG antibodies were positive for BP180 but not for BP230 [11,13]. Otherwise, the target antigen of BPP has not yet been clearly established, which is why clinical and histopathological findings are crucial in diagnosing this disease [2,4,14].

Dermoscopy can be helpful in the diagnosis and differentiation of BPP from other dermatoses causing cicatricial alopecia. Data about dermoscopic signs of BPP are scarce. The main dermoscopic differentiating signs of BPP are the presence of white lamellar structures and yellow dots with a whitish halo (“fried-egg sign”), corresponding to the detached epidermis of the interfollicular area and follicular openings, respectively, resulting in scarring and permanent hair follicles loss in parietal and temporal areas [6]. The main dermoscopic differential diagnosis of BPP is summarized in Table 1.

**Table 1:** Differential diagnosis of Brunsting-Perry pemphigoid This tabulated information compares dermoscopy signs between Lichen planopilaris, Pseudopelade of Brocq, and Brunsting-Perry pemphigoid based on data compiled from Rudnicka et al. [6].

	Lichen planopilaris	Pseudopelade of Brocq	Brunsting-Perry pemphigoid
Pattern	“Strawberry ice cream” – on a recent onset	“Footprints in the snow”	“Fried-egg sign”
Perifollicular inflammation	Red halos around hair-bearing follicles	No	Large yellow dots with whitish halo
Perifollicular collar scaling	Silver-white tubular scaling along the hair	No	Mild scaling
Perifollicular fibrosis	White halos	Follicles are absent	Follicles are absent
Interfollicular area	Smooth and slightly glossy	Smooth white areas	White lamellar structures
Violaceous or violet-brown areas	Common in inflammatory lesions	No	No
Cicatricial changes	Smooth and reflective pink	Smooth and white	White confluent areas on a milky red background
Vascular network	Elongated, small, coiled, and dotted perifollicular vessels	No	Elongated serpentine, dotted vessels with a whitish halo
Blisters and erosions	No	No	Present

As with other pemphigoid diseases, BPP is usually treated with topical and/or systemic steroids. In severe or resistant cases, diaminodiphenyl sulfone (dapsone), azathioprine and cyclophosphamide, doxycycline, niceritrol, and nicotinamide may be an additional treatment option [7]. In a single case of refractory to multiple conventional therapies, BPP was successfully treated with a dupilumab regimen of 300 mg every two weeks [15]. In another case, BPP was cleared by dupilumab after the two-week interval doses, with maintenance of the result by monthly doses for 6 months [10].

## Conclusions

We reported a rare case of BPP causing cicatricial alopecia and discussed the criteria of its diagnosis based on the reported literature on similar cases. Diagnosis of BPP is rare and should be suggested in cases of blistering and scarring, mainly limited to the head, scalp, and neck area, often with permanent hair follicle loss in the parietal and temporal areas. A dermoscopy picture of the “fried-egg sign” helps to differentiate BPP from other dermatoses causing cicatricial alopecia. Histopathology presents a subepidermal blister with dermal inflammation, which immunopathologically shows IgG, IgM, and often C3 deposits linearly distributed along the basement membrane zone. Treatment by topical or intralesional steroids is a primary choice, but the use of anti-inflammatory biologics is considered to be effective in refractory cases.
